# Virtual Morris Water Task: Procedures and Protocols for the Assessment of Spatial Navigation and Memory

**DOI:** 10.1002/cpz1.70340

**Published:** 2026-02-27

**Authors:** Conor Thornberry, Jose M. Cimadevilla, Sean Commins

**Affiliations:** ^1^ Trinity College Institute of Neuroscience Trinity College Dublin Dublin Ireland; ^2^ School of Psychology Trinity College Dublin Dublin Ireland; ^3^ Department of Psychology and Health Research Center University of Almería Almería Spain; ^4^ Department of Psychology Maynooth University, Co. Kildare Kildare Ireland

**Keywords:** learning, memory, Morris water maze, spatial navigation, virtual reality

## Abstract

The original Morris water maze has been coined the “gold standard” task for examining spatial navigation in animals. The general procedure of the maze involves a circular pool filled approximately halfway with water. An animal is then tasked with locating and recalling the position of a hidden “platform,” which is submerged below the water surface in a fixed location. The platform has minimal visual presence in the pool, meaning the location of the platform must be found, learned, and recalled from memory. Recently, the task has been translated using virtual reality for use with humans (virtual Morris water task) to investigate similar cognitive mechanisms examined using the animal version of the task. However, there are multiple variations of the virtual Morris water task scattered across the human literature. These versions vary in both environmental design (e.g., different shaped arenas or platform sizes) and testing procedures (e.g., 1‐min trial times or no intertrial intervals), which influence a person's ability to perform the task. While the virtual version of this task possesses the same potential to become the “gold standard” for examining spatial cognition in humans, comparing and replicating results across research labs has been incredibly difficult due the lack of standardized procedures and protocols. In this paper, we present protocols to help with the standardization of this task. We recommend practices and procedures for researching specific cognitive processes in humans, as well as reporting guidelines, recommended analyses, and expected results. © 2026 The Author(s). *Current Protocols* published by Wiley Periodicals LLC.

## BACKGROUND

The original water maze developed by Richard Morris in 1981 has been at the forefront of learning and memory research for many years (Morris, 1984; Morris, 1981; Morris et al., 1982; Vorhees & Williams, 2006). The task is a simple and effective test that is primarily used to examine spatial learning and recall in rodents (D'Hooge & De Deyn, 2001). The popularity of the task was cemented when it was shown to be hippocampal‐dependent (Morris et al., 1982). Furthermore, it is sensitive to age (Frick et al., 1995), sex (Cimadevilla et al., 2004), environmental (Cao et al., 2008; Farina et al., 2015), behavioral (Fenton et al., 1994; Hölscher, 1999), neural (Broadbent et al., 2006; Packard & McGaugh, 1992), immediate early gene (Farina & Commins, 2020; Shires & Aggleton, 2008), and pharmacological (Morris et al., 1982; Skarsfeldt, 1996) manipulations. In addition, its use across multiple species and with models of different diseases and disorders, such as Alzheimer's disease (Bromley‐Brits et al., 2011; Commins & Kirby, 2019), Parkinson's disease (Pothakos et al., 2009), and epilepsy (Inostroza et al., 2011), has made the Morris water maze (MWM) the “gold standard” tool for animal learning, memory, and navigation research over the last 40 years.

The water maze task typically involves a circular pool (1–2 m in diameter, species‐dependent) filled partway with water that is often rendered opaque (Nunez, 2008). The rodent is tasked with escaping the water by finding a hidden platform, located just under the water's surface. As the animal cannot see the platform directly, it must learn and subsequently recall its location. Depending on the experimental manipulation, animals may rely on various strategies to find the hidden platform, including distal landmarks (Chapillon, 1999), beacons (Timberlake et al., 2007), audio cues, or their own trajectory (Gehring et al., 2015), among others. The maze provides a highly controlled environment for landmark manipulation, behavioral observation, and lesion studies (Barry & Commins, 2019; de Bruin et al., 1994; Miyoshi et al., 2012; Morris, 1984). With the advancement of technology [especially the growth of virtual reality (VR)], the interest in directly translating animal findings to humans and the need for clinical tools, a plethora of VR spatial navigation tasks have been developed over recent years including virtual towns (Newman et al., 2007), virtual islands (Piper et al., 2010), and large open environments (Wiener et al., 2020). However, in a recent review, Thornberry et al. (2021) report that one of the most popular tasks used to test human spatial learning and memory was the virtual water maze (VWM) task, originally developed by Astur et al. (1998) and modeled on the traditional MWM (Morris, 1984; Morris, 1981). Despite the task's popularity, Thornberry et al. (2021) highlighted the lack of standardization in its use, particularly with respect to the environmental setup and procedures. For example, there are inconsistencies in terms of the arena size and number of trials or trial length, ranging from a 30‐s trial with an intertrial interval (ITI) of 15 s (Antonova et al., 2011) to a 180‐s trial with no ITI (Kallai et al., 2005). Such inconsistencies in the use of the VWM make replication and direct comparisons of experimental results between laboratories extremely difficult. This is particularly problematic for examining clinical populations, especially given that spatial tasks, including the virtual Morris water task, have the potential to become early diagnostic tools for dementia and Alzheimer's disease (Coughlan et al., 2018; Laczó et al., 2012). Accepting that variations in protocols exist across laboratories with respect to the rodent water maze task, a comprehensive attempt to standardize procedures for animal research was proposed by Vorhees and Williams (2006). With this in mind, we attempt to propose a similar set of protocols for use with the human VWM task.

## TEST PROTOCOLS

### Spatial Learning

Place or spatial learning is the most basic procedure for both the MWM (Vorhees & Williams, 2006) and VWM tasks. (Hamilton et al., 2002). Participants are tasked with exploring an open area in search of a hidden goal location. Once found, participants must try to recall this location, typically using environmental landmarks (Mueller et al. 2008) and navigate back to the goal location in each subsequent trial. As with MWM studies (Alcalá et al., 2020; de Bruin et al., 1994; Hölscher, 1999; Miyoshi et al., 2012; Morris, 1984), most protocols using the VWM use semi‐random trials starting at the cardinal points of the arena (N, S, E, and W), or even add mid‐cardinal points (NE, NW, SE, and SW) (D'Archangel et al., 2022; de Castell et al., 2015). Although the use of these starting positions generates long and short paths (see Vorhees & Williams, 2006, for possible solutions to this), the rationale behind using multiple starting points is to prevent procedural learning and promote the use of environmental landmarks and the formulation of a cognitive map (Iaria et al., 2009).

In nonhuman animal studies, acquisition training typically occurs over several days, with multiple trials per day (Baldi et al., 2005; Gulinello et al., 2009; Wenk, 2004). However, it may be difficult and impractical to repeatedly train and test humans in a virtual environment across multiple days. A single training session for both patients and nonpatients alike is effective (Commins et al., 2020; Deery & Commins, 2023; Kolarik et al., 2016). Furthermore, many studies train participants using a single block of trials (Goodrich‐Hunsaker et al., 2010); multiple blocks with a reduced number of trials within each block have also been used successfully (Astur et al., 1998; Newhouse et al., 2007; Woolley et al., 2010). There is no agreement in the VWM literature regarding the total *number of trials*. For example, some participants receive 18 trials (6 blocks of 3 trials) (Hamilton et al., 2002), some receive 28 trials (7 blocks of 4 trials each) (Driscoll et al., 2005), and others just 6 trials (single block) (Herting & Nagel, 2012); see Thornberry et al. (2021, p10) for details. Although many studies have shown successful spatial learning with a small number of trials (Müller et al., 2018), 12 trials is the minimum number suggested in the animal literature (Nunez, 2008) and maybe useful for the VWM too, especially given that humans may learn the task at the same rate, if not faster than animals (Schoenfeld et al., 2017; Thornberry et al., 2021). In addition, 12 trials may allow asymptotic learning to occur (Daugherty et al., 2015) and would also facilitate combinations of starting points, allowing for 3 rotations of 4 starting positions.

*Trial length* in both the animal and human literature also varies (D'Hooge & De Deyn, 2001; Vorhees & Williams, 2006) but 60–180 s is a popular trial length mentioned in the current human literature (see Table 3, Thornberry et al., 2021), but this may be contingent on the participant group, size of the arena, and experimental procedure. For example, patients and individuals in an MRI scanner may need an increased trial time and/or a reduction in the overall trial number (Folley et al., 2010). Although it may be useful for older adults and patient groups to partake in practice trials (described below), increasing either the number of trials or the time to find the goal in each learning trial may also prove useful. For example, Astur et al. (2002) successfully used 20 trials of 60 s duration when they examined spatial learning using the VWM in patients with hippocampal damage. Alternatively, it may be useful to incorporate more landmarks or beacons so that participants can perform the task as efficiently as possible. For example, this is a common method for older adults or those with specific diseases that would cause delayed learning or motor movement (Cánovas et al., 2009; Reynolds et al., 2019). Researchers may also be limited by the software they are using and the timings available.

Using an ITI between learning trials may also be useful to facilitate learning as well as provide a break for participants (Commins et al., 2003; Karpicke & Bauernschmidt, 2011). The length of this ITI again varies in the VWM literature, with some researchers not implementing any ITI (Kallai et al., 2005) and others having an ITI of up to 30 s (Sandstrom et al., 1998). In the animal version of the task, an ITI between 5 and 15 s is commonly used (Vorhees & Williams, 2006). Allowing 10 s between each trial (while the participant waits in the target area) is effective in the human literature too (Hamilton et al., 2002; Hamilton et al., 2003). However, the choice of time may depend on the study and the participant group. For example, a shorter ITI could be used (Astur et al., 2004) for a larger number of learning trials to counteract boredom, particularly for good learners.

In the MWM task, if a nonhuman animal does not find the hidden platform in a given trial, it is led to it by the researcher (McGauran et al., 2005). This is done to indicate the presence of a goal, to promote learning the goal location rather than the route, and to encourage the association between landmarks and the platform. Movement to the target in unsuccessful VWM trials may be done by teleporting the participant to the target area (Commins et al., 2020), allowing the platform to become visible with on‐screen instructions to “swim” to it (Astur et al., 2004) or activating a “free‐movement” mode, whereby the participant can be guided by the researcher (Goodrich‐Hunsaker et al., 2010). Once at the target site, time is given for the participant to look around; this time varies, but typically 10 s has been reported for MWM and VWM (Ben‐Zeev et al., 2020).

### Spatial Recall

A single‐probe trial is often used following learning to assess spatial memory and recall ability in animal and human literature (Barnhart et al., 2015; Buckley & Bast, 2018; Dobbels et al., 2020; Piber et al., 2018). During a probe trial, the previously learned goal location (e.g., escape platform or target) is removed or made unavailable, and participants are allowed to freely search the environment. Spatial memory is indexed by search behavior relative to the former goal location, such as time spent in the target region, providing an assessment of recall that is independent of task performance and feedback (see D'Hooge and De Deyn, 2001 for animals and Buckley and Bast, 2018 for humans). Often, a period of time is provided between the final trial of learning and the recall test to ensure that the spatial memory being examined is reference memory, that is, long‐term stable memory for a consistent goal location learned over repeated exposure (Baldi et al., 2005; Ekstrom & Hill, 2023). This contrasts with spatial working memory, which relies on short‐term retention of trial‐specific information such as the most recent goal location (see below). For example, a 5‐ (Jiang et al., 2022), 10‐ (Bolding & Rudy, 2006), or 30‐min gap (Schoenfeld et al., 2014) between the final learning trial and the probe trial has been used successfully in the VWM. A longer period, such as 24 h, may be useful to examine long‐term memory (Baldi et al., 2005; Gulinello et al., 2009) and/or the impact of sleep (Samanta et al., 2021; Schapiro et al., 2019). Some researchers ask participants to carry out alternative tasks (e.g., pen and paper or another computer‐based task) during the gap period to remove the focus away from the VWM task (Gulinello et al., 2009). The probe trial duration typically used is similar to the time allowed for each learning trial (e.g., 60 s, Thornberry and Commins, 2024). Although a 60‐s probe trial is widely used in VMW literature (Astur et al., 2002; Maei et al., 2009; Nunez, 2008), longer times have also been used (e.g., 120 s, Schoenfeld et al., 2017). However, close consideration should be given to the first 30 s, as during this time, the participants tend to head directly toward the goal location before searching in alternative locations (Luna & Martínez, 2015; McGauran et al., 2004).

### Spatial Working Memory

Working memory refers to a limited‐capacity system responsible for the temporary maintenance and manipulation of information to support cognitive tasks (Baddeley & Hitch, 1994; Hitch et al., 2025). In spatial tasks, working memory typically refers to short‐term retention of trial‐specific spatial information (e.g., a spatial location or relation between objects) that must be updated across a task, typically during navigation (Blacker et al., 2017).

Spatial working memory and its neural substrates can also be assessed using both the MWM and VWM (see Rodriguez, 2010). Vorhees & Williams (2006) suggested a protocol for the MWM that consists of two trials: an encoding trial and a test trial. In the encoding phase, the animal is required to find the hidden target through trial and error. Once found and following a 15‐s break, the test phase begins. The animal is again tasked with finding the target, but in this phase, the animal should be quicker and take a shorter path. The time saving between the two trials is used to measure working memory. This protocol is repeated (typically over days), but the target is moved to a new location to prevent the formation of a stable reference memory for the goal location. A slightly modified version of the task was used by Rodriguez (2010) in the VWM. Again, two trials are used. In the encoding trial, the participant starts in a random location and must move toward a *visible* target within a set period of time. Once found, the participant is moved to a new random location. In the test trial, participants are tasked with finding the same target location that is now rendered invisible. A time limit of 15 s for the encoding trial and 20 s for the test trial was given. The number of times the correct location was found was used as a measure of working memory. As in the animal version, the goal and starting point are changed for each set of trials. A sample protocol (Rodriguez, 2010) is provided in Table 1. Researchers may use a unique starting position (e.g., S) and a hidden goal location (e.g., NE) for Trial 1, which are then repeated for Trial 2 but in the absence of the target platform. This setup can then be changed for every 2 consecutive trials. The percentage time (of a set time, such as 20 s) in the target quadrant (area) for the test trial can be used to assess working memory. The number of times the protocol is repeated varies depending on the experiment. For example, Rodriguez (2010) imaged participants who had at least 7 correct trials for the experiment.

**Table 1:** Examples of platform locations and starting positions for each type of protocol outlined above. The total number of trials displayed is 4, and these can be alternated and repeated to meet the planned number of trials for the experiment. A probe is a recall trial to examine recall and is labelled as such below.

Standard learning	Trial 1	Trial 2	Trial 3	Trial 4
Position of platform (center of)	NE	NE	NE	NE
Starting position	S	N	W	E
Standard recall	Probe 1			
No platform available	‐			
Starting position	SE			
Working memory	Session 1		Session 2	
	Trial 1	Probe 1	Trial 1	Probe 1
Position of platform (center of)	NE	‐	NW	‐
Starting position	S	S	W	W
Delayed matching‐to‐place (DMP)	Session 1			
	Trial 1	Trial 2	Trial 3	Trial 4
Position of platform (center of)	NE	NE	NE	NE
Starting position	W	E	N	S
	Session 2			
Position of platform (center of)	NW	Probe 1	NW	NW
Starting position	S	N	E	W
Spatial reversal	Trial 1	Trial 2	Trial 3	Trial 4
Position of platform (After spatial learning with platform in NE)	SW	SW	SW	SW
Starting position	N	S	E	W

An alternative solution for working memory testing is using a delayed matching‐to‐place (DMP) protocol, used in a virtual environment by Buckley and Bast (2018). The authors used 24 total trials, with a hidden goal location changing every 4 trials. Participants carried out the task in blocks of 4 trials, starting from cardinal locations in random order. Trials were started by the participant pressing “enter” after the previous trial. Participants were given 120 s per trial. Only 2 of the 24 trials were probe trials. Probe trials terminated after 60 s, giving a “keep looking” message before the next learning trial. Between goal location changes, a “new location” message was displayed for 3 s before the first trial. This would provide insight into the ability to rapidly form and maintain representations about the locations of landmarks and goals (Buckley & Bast, 2018; Glöckner et al., 2021; Morris et al., 1986). While not exactly examining working *memory* as such, the protocol may investigate the formation of longer‐term representations based on the use of working memory to solve rapidly presented probe trials, similar to the previous paradigm. Critically, these paradigms examine working memory by requiring short‐term maintenance and rapid updating of trial‐specific goal locations over *brief* delays, while preventing reliance on stable, long‐term reference memory representations.

### Reversal Learning

Reversal learning refers to the ability to update previously learned information when task contingencies change (Izquierdo et al., 2017), such as having to forget an old location and learn a new one. Reversal learning is used to examine cognitive flexibility and can help us understand how we discriminate between locations. This procedure is also regularly used for assessing lesions or pharmacological impacts on learning (Hoh et al., 1999) and has been successful in animals (Walsh et al., 2011) and the VWM (Schoenfeld et al., 2014). Reversal paradigms can also help understand brain function in processes not directly related to spatial learning or memory, such as attention and decision‐making (Izquierdo et al., 2017; Shah et al., 2018). A reversal phase may also improve overall learning performance, which may be useful in patients or older adult populations (Alcalá et al., 2020).

Typically, a learning phase should occur (as outlined above). The participant should be allowed a small break before starting the reversal learning session. To examine reversal learning, immediately following the learning trials, the target would be moved to another quadrant (normally the opposite; Vorhees & Williams, 2006) and the participant is then required to find the target again. For the reversal trials, the ITI length and the environment layout typically remain the same as those used for the learning trials. The number of trials, however, can vary. For example, Schoenfeld et al. (2014) used a further 6 reversal learning trials after the initial 12 learning trials. The example in Table 1 uses 4 trials, 1 trial from each of the cardinal starting positions. A reversal probe trial can also be given at the end of the learning session and again at the end of the reversal trials (Fajnerová et al., 2014).

### Cued Learning

Cued learning refers to navigation that is guided by explicit environmental cues signaling the location of a goal, rather than relying on spatial memory (Dringenberg et al., 2001). Though cued learning may be used as an experimental control, as mentioned by Vorhees & Williams (2006) and Rodriguez (2010), there is the opportunity to utilize the task as a form of egocentric and procedural learning (Packard & McGaugh, 1992) to assess saliency models or landmark dependence during navigation (Chamizo et al., 2006; Commins & Fey, 2019; Farina et al., 2015; Lee & Spelke, 2010) or as a comparison of different navigational strategies (Livingstone‐Lee et al., 2014). In both the MWM and VWM tasks, the participant is simply required to head toward the beacon, which is (virtually) located on or beside the goal location (Waller & Lippa, 2007). Alternatively, the target itself remains visible, and the participant simply moves toward it (Ferguson et al., 2019; Hamilton et al., 2003). The time taken to reach the target is recorded. Typically, the target and/or the starting position is moved to a new location for every trial to prevent place or response learning (Ferguson et al., 2019; Livingstone & Skelton, 2007). The number of trials may again vary depending on the experimental aim and/or participant profile; for example, Ferguson et al. (2019) used 4 trials with the visible target moving further from the start point with each trial, while Hamilton et al. (2003) used just 2 trials for children with fetal alcohol syndrome. As a practice phase, cued trials may be useful to determine the impacts of visual or motor deficits, motivational issues, as well as allowing participants to become familiar with the computer hardware and software. For example, Reynolds et al. (2019) provided practice trials to younger and older participants who were instructed to make their way toward 4 visible objects located in the virtual arena. A minimum of 4 trials would allow participants to explore the environment from all 4 cardinal points. The visible platform placement during practice or cued learning trials should not match the novel location used for invisible learning trials.

### Further Control Procedures

It is particularly important to control for non‐learning effects (such as movement, visual, and motivation) during task acquisition, especially when trying to correlate neural markers (e.g., fMRI, EEG, and MEG) to learning and memory performance; these are seldom included within the experimental design. As previously discussed in Thornberry et al. (2021) and Vorhees & Williams (2006), common control procedures for the MWM task include allowing animals to swim freely around the pool for the same period as the learning counterparts (Shires & Aggleton, 2008). Others used cued or beacon learning groups, but this “control” still involves a form of learning (Barry & Commins, 2019; Wolbers & Wiener, 2014). VWM approaches have been similar, using free movement conditions for the same length of time as learning trials or cued learning (Goodrich‐Hunsaker et al., 2010). Similarly, in a recent study that analyzed EEG correlates during spatial learning and recall, Thornberry & Commins (2024) used a separate non‐learning group that was required to explore the same environment as a learning group but without a target being present. Each trial in the non‐learning group was time‐matched to the mean time taken by the learning group to reach the target. Furthermore, any control group should be matched for age, gender, and other demographics.

## METHODS AND MATERIALS

### Headsets and Desktop

VR headsets (such as the HTC Vive or Oculus Rift) should be used in a large, open environment, allowing participants to move safely.

*CAUTION*: The safety manual of the hardware manufacturer should be followed. All safety devices should be engaged, including head straps and wrist straps, as recommended by the manufacturer. Careful attention should be given to vulnerable participants, including older adults, patients, and children who may be more susceptible to motion sickness or have balance issues.

If using a virtual 2D desktop system, a quiet location and a fast, reactive computer should be used. Joysticks may be preferred (for their ease of use) over gamepads or mice and keyboards; however, joysticks may favor those with video game experience.

*CAUTION*: Familiarity with gaming technology and frequency of use should be considered and/or measured before commencing a VR or desktop experiment.

### The Virtual Maze Arena

The arena should be circular to match the original MWM for simplicity and translatability. Different‐shaped arenas may be used when examining the effect of geometry on learning (Horne & Pearce, 2009). The size of the arena (in virtual meters) and its traversal time should be noted.

*CAUTION*: The larger the arena, the more difficult it is to navigate and find the hidden target (Commins et al., 2020). However, a traversal time (along the diameter) of 10–15 s is effective (Commins et al., 2020).

**Figure 1:**
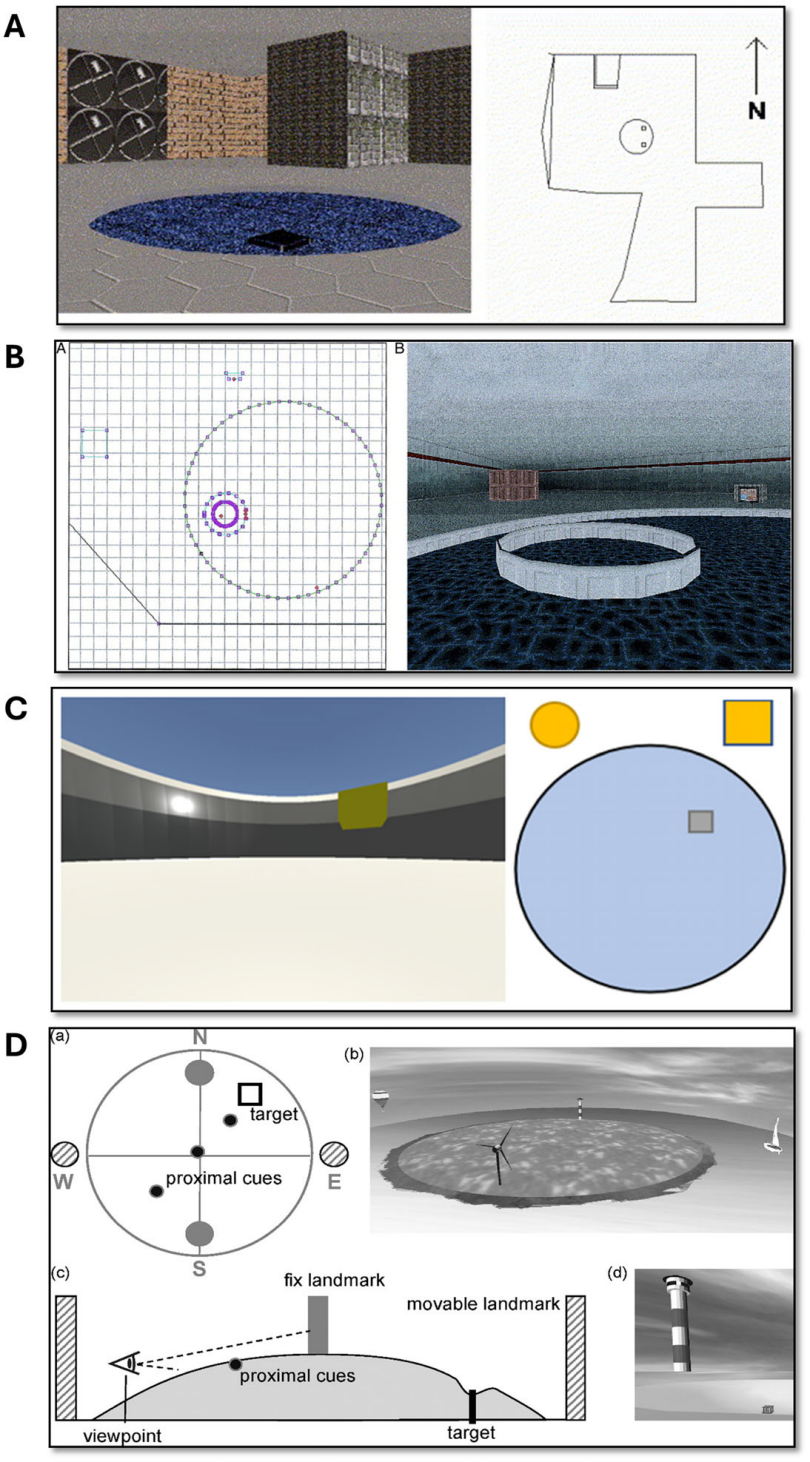
Examples of VWM arena design and publication of specific environment details, which includes pool size relative to external environment **(A)** (Astur et al., 2004), platform size relative to pool **(B)** (Sandstrom et al., 1998), cue positions and appearance **(C)** (Commins et al., 2020), and diagrams and images of all relative information **(D)** (Schoenfeld et al., 2010).

The arena should be enclosed by a boundary wall. Distal landmarks may be located near the boundary (Redhead & Hamilton, 2009), on the boundary (Commins & Fey, 2019), or outside the boundary (Sandstrom et al., 1998). Figures 1b and 1c show examples. Virtual landmarks located within the arena, allowing for participant interaction, may be used as proximal cues or beacons (particularly if located on or beside the goal; see above).

The bounded arena may be located within a room, whereby the room and its walls may act as distal cues (Figure 1a and 1b).

### The Goal/Target/Platform

The goal should be located within the arena. This may be located anywhere but is typically located in the middle of one of the four quadrants of the arena (NE, NW, SE, or SW; Newhouse et al., 2007; Vorhees & Williams, 2024). The goal should remain invisible (for spatial learning) until it is found by the participant.

*CAUTION*: A small target in a large arena may impact learning; this has been observed in the animal literature (Vorhees & Williams, 2006; Vorhees & Williams, 2014a, 2014b). A target size between 10% and 20% of the total arena seems to be common for VWM experiments (see Thornberry et al., 2021, for details). Reporting the target as a percentage of the environment's total virtual size rather than an actual size allows for direct comparison between labs.

### Landmarks

Simple and easy‐to‐define landmarks may be preferred over others. Cues should be easily verbalized, as more abstract cues may impair performance (Barkas et al., 2010). Large shapes with distinctive features or well‐known objects have been successfully used in the VMW as landmarks (see Figure 1 for examples). As mentioned, landmarks may be positioned within, on, or outside the arena, depending on their use as proximal or distal cues.

The number, location, and salience of landmarks may impact spatial learning. However, participants can successfully navigate with a single one (Cánovas et al., 2011) and landmarks located further from the goal may impair performance compared to those located closer to the target in both animal and human studies (Chamizo et al., 2006; Commins et al., 2020).

*NOTE*: There is emerging evidence that individual differences, including sex, may play a role in task performance (Nazareth et al., 2019). Recent literature seems to indicate that individual differences are not about strategy but about the use of landmarks (Kolarik et al., 2016; Liu & Borisyuk, 2024; Liu et al., 2022; West et al., 2023). This should be considered when selecting landmarks (types and numbers) and their positions (Astur et al., 1998; Astur et al., 2004; Boone et al., 2018; Chai & Jacobs, 2009; Padilla et al., 2017).

*NOTE*: Full dimensions of the arena, along with the size, number, type, and location of landmarks, should be provided. In addition, the provision of screenshots may be useful to allow for further visualization.

### Instructions and Motivation

1. Instructions should be presented on the screen *as well as* described by the researcher before beginning the task; this may control for possible gender differences (Nazareth et al., 2019).
2. A countdown between trials may be considered to help participants prepare for the next trial.
3. The original water maze task had its own motivator with the presence of water. This is not present in the human version of the task. As such, motivational indicators may be used, including skill‐based level ratings presented at the end of each trial (Coutrot et al., 2019) or a high‐score system with a live points tally. Feedback when the platform is found may also be sufficient (Commins et al., 2020), with a challenge prompt appearing on the screen, such as *“Try again”* (see Tuena et al., 2023) or feedback after particular events (Baker & Holroyd, 2008).

## PROCEDURE

### Practice Phase

1. Participants should be introduced to the experimental space by the researcher (e.g., desktop, EEG recording suite, and VR room).
2. The participants should be introduced to the virtual environment, and the objective of the task should be explained by the researcher. The controls (mouse and keyboard, joystick, and Oculus controllers) should also be explained in sufficient detail.
3. The researcher should remain in the room with the participants and initiate the training/practice phase of trials. We recommend that the target itself remain visible and the participant simply move toward it (see Cued Learning). The target and starting position should be moved to a new location after each trial to allow for flexible practice of software controls and understanding of the procedure.
4. We recommend that 4 trials should be used at a minimum, with the starting point of each being 1 of the 4 cardinal points of the arena. The target should *not* be in the same location as it was during the learning phase.
5. On‐screen instructions should display a message “Please move to the visible target.” The time should match the chosen experimental time limit. There should be *no landmarks* during this phase to prevent improved performance for the learning phase.
6. Participants should be queried after the 4 trials as to whether they are comfortable using the controls and whether they understand the task. Should the participants request more practice, steps 1 to 6 may be repeated.
7. Should participants be comfortable but have been unsuccessful during their practice phase (i.e., could not locate the visible platform within the time limit in repeated trials) or vice versa, researchers should consider their exclusion prior to implementing the learning phase explained below.

### Basic Spatial Learning Procedure

1. Participants are set up in an experimental space (if using VR or neural recording systems) and/or positioned in a room free from distraction in front of a computer (if using a desktop).
2. The participants view the environment from a first‐person perspective and start from 1 of 4 cardinal points in the arena (see above). Instructions may be displayed on screen and/or relayed by the researcher.
3. The participants navigate using the virtual controllers, joystick or keyboard, and mouse controls through the environment. The time taken to reach the hidden target is recorded; the time starts as soon as the participants begin moving and ends when the participants reach the target or when the trial time has elapsed (we recommend at least 1 min; see above). If possible, the path length should also be recorded. These are typically recorded by the software.
4. Should the participants not locate the hidden goal location within the trial time limit, they may be guided to the goal.
5. The participants may have an ITI with a minimum of 5% of the total trial time (e.g., for 60 s, the ITI should be a minimum of 3 s) before starting the next trial at a new location. Repeat steps 2 to 4 until the preset number of trials has been reached (e.g., 12 trials; see above). Shorter ITIs may be used if the environment is particularly easy to navigate, to ensure focus and engagement during the task. Longer ITIs should be avoided unless the task is incredibly challenging or the trial duration is extensive (e.g., for particular populations).
6. Researchers should record and report the time spent on the target during the ITI and may include it in subsequent analyses of learning. This is particularly important for participants who did not successfully locate the platform within their trial time.

### Spatial Recall/Probe Trial

**Figure 2:**
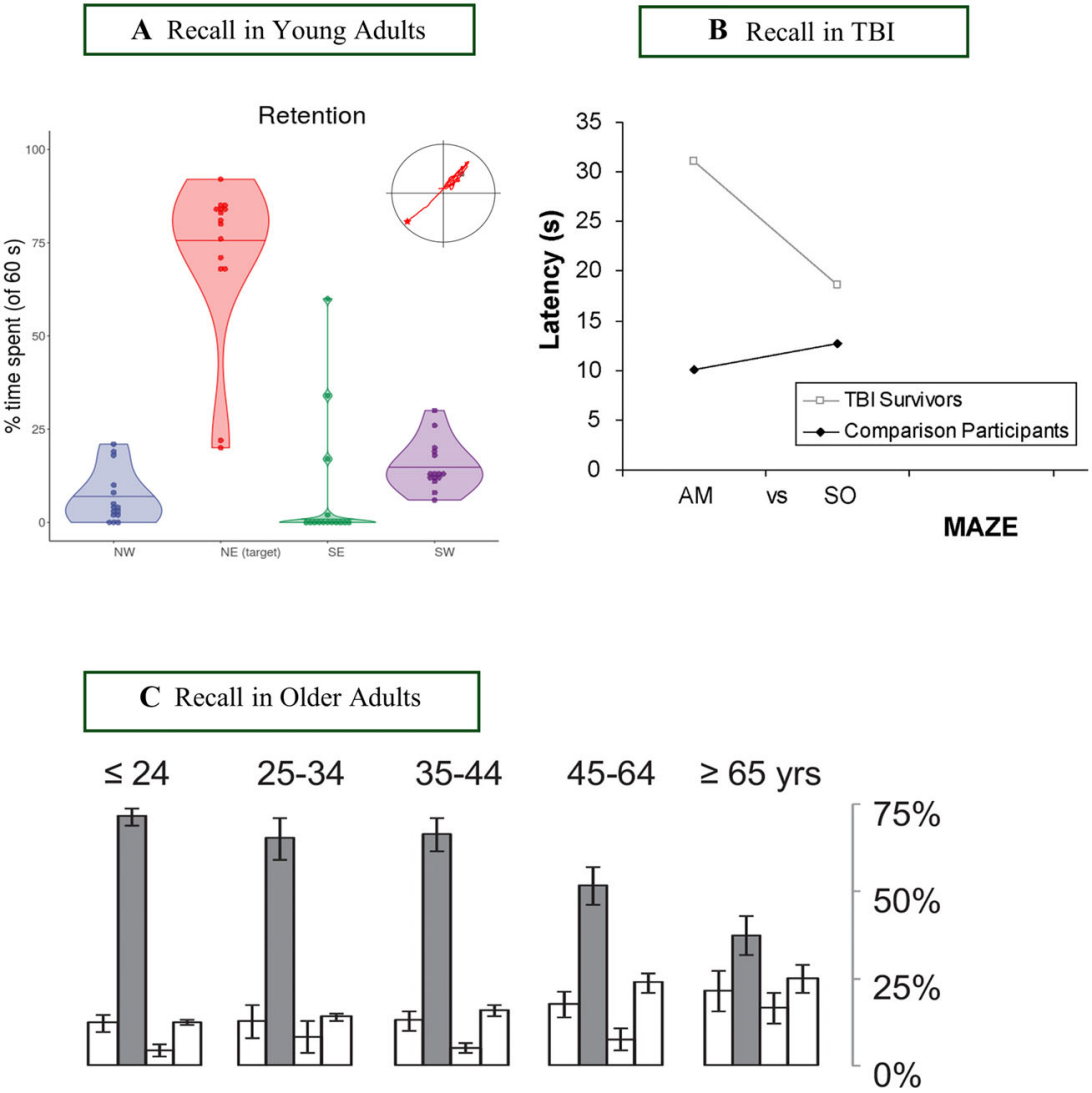
Examples of recall ability in younger adults **(A)** (Commins et al., 2020), traumatic brain injury (TBI) patients **(B)** (Livingstone & Skelton, 2007); (dwell time was higher for TBI patients compared to controls), and older adults **(C)** (Schoenfeld et al., 2014); the grey bar is the target quadrant.

*NOTE*: At chance level, participants would spend 25% on each of the quadrants. We typically see recall percentages of 70% or higher (e.g., Figure 2A). However, this can be lower for patient populations (Figure 2B) and vary across age (Figure 2C).

7. Following completion of the assigned number of learning trials, the goal is removed.
8. Participants are then given a break between learning and recall trials (we recommend 5–10 min, or longer if testing probe remote memory; see above).
9. *Alternatively*, researchers may opt for a shorter probe trial break, e.g., less than 1 min after learning. This is termed as an “immediate recall” in the literature (Schoenfeld et al., 2014). This may map better onto memory‐related pen‐and‐paper cognitive tasks such as the Rey Auditory Verbal Learning Test (RAVLT; Schmidt, 1996) that use similar immediate recall periods to examine memory retention.
10. Begin the recall trial from a new start position (not used previously; e.g., a point between 2 cardinal points). We suggest that researchers start the recall trial 180° from the original platform position (Mehta et al., 2021; Vorhees & Williams, 2006). The new location is to ensure that participants are recalling the location of the goal, and not a previously learned search path. It should *not* be one of the cardinal points.
11. The probe trial is typically the same length as one of the learning trials (e.g., 60 s). The percentage of time spent in each quadrant (NW, NE, SW, and SE), particularly the goal quadrant, is typically used to measure recall (see Figure 2). Other measures, including the time/distance to the location or goal crossings, may also be used (Maei et al., 2009). These measures may depend on the software used.

### Spatial Working Memory

Each session consists of 2 trials, 1 learning trial and 1 probe trial, each trial being at least 1 min (as recommended above). It is also possible to include blocks of 4 trials with alternating platform positions (DMP, see Buckley & Bast, 2018).

The trial design and starting positions can be organized as discussed in the protocols section (see Table 1 for examples). The participant must navigate quickly to a visible platform and learn its location. The second trial is an invisible platform probe trial (see above) based on the previous trial setup, starting position, and cue layout.

A longer ITI may be given due to the complexity of the task, about 15–30 s (approximately 25% of the total trial time), based on the previous discussion (also see Commins et al., 2003).

The platform should be moved between each pair of trials, and the starting direction should be changed between sets of trials, to prevent reliance on motor movements or route learning.

## RECOMMENDED STATISTICAL ANALYSES

Power calculations to estimate required sample sizes are very important for reproducibility (Bishop, 2020). As Vorhees and Williams (2021) rightfully argue, sample size calculations are often limited due to animal agency regulations. However, for human studies, simple *a priori* sample size and power requirements should be reported; these have recently proved valuable for VWM paradigms with complex measurements (Schoenfeld et al., 2017; Thornberry et al., 2023). Many open‐access statistical packages offer power calculation tools, including *R* [https://www.r‐project.org/; R Core Team (2013)] and G*Power (http://www.gpower.hhu.de/; Faul et al., 2007). Some studies have used sample size calculations with animals (Barry & Commins, 2019; Young et al., 2009). For tasks with varying protocols, such as the VWM, researchers need to compare power and sample sizes across different versions of the virtual task (Schoenfeld et al., 2017). Reporting these data will help improve the current replication crisis in psychological science (Shrout & Rodgers, 2018). It will also address the current lack of openness and reliability in the neurosciences (Huber et al., 2019).

At a minimum, group means and standard errors (SEM) should be averaged over each learning trial and presented. Alternatively, trials may be averaged in blocks (e.g., blocks of 4 trials; Zhong et al., 2017) and plotted as block means (± SEMs). Depending on the protocol, groups should be compared appropriately. For example, mixed between‐within analysis of variance (ANOVA) should be used to analyze differences between groups across the trials, with particular focus on differences between early and late learning trials (Trial 1 and Trial 2 vs. Trial 11 and Trial 12). Group (for example, sex) should be the “between” factor, with trial being the “within” factor. Appropriate *post‐hoc* tests (e.g., Bonferroni‐correct *t*‐tests for within‐group comparisons and Tukey for between‐group comparisons) should be used to follow up on main effects. For data visualization, group means should be reported, but individual data points should be jittered and displayed on graphs (Weissgerber et al., 2015). This is particularly useful for the critical evaluation of continuous, small sample data. Learning data from navigation tasks typically produces high levels of individual variability (Coutrot et al., 2019) and should be examined appropriately if these concepts are of interest using more advanced techniques [e.g., Generalized linear mixed models (GLMMs); see Commins et al., 2023]. For probe trials, group means and SEMs should be compared using appropriate group‐level statistics (e.g., ANOVA) based on typical measurements such as time and distance in the target quadrant.

We would recommend that researchers make as much of their VWM research openly available as possible. This should include depositing both raw and manipulated data in online repositories (such as https://osf.io/) with descriptions of all analyses performed and relevant code (if applicable). If possible, manuscripts should be preregistered to reduce bias, making the work more reproducible by other researchers with an easily accessible and transparent process for each statistical and methodological decision (Crüwell et al., 2019). Papers should be published as preprints on sites such as bioRxiv or PsyArXiv (complete list at http://v2.sherpa.ac.uk/opendoar/) and/or made open access after publication if possible (Crüwell et al., 2019). Furthermore, if the VWM task itself can be made open access for other researchers to use, this should be a priority (see NavWell; https://navwell.cs.nuim.ie/home). Virtual tasks can be costly and can even be a barrier to open‐access data sharing. By enacting an open science policy with VWM tasks, some consistency across research groups should naturally emerge.

## TRANSPARENCY AND OPENNESS

All data that will follow have been made publicly available via OSF and can be accessed at www.osf.io/ch5rn. The analysis code for this study, used to produce Figure 5 below, is also available in this repository. We intend for researchers to examine this dataset, to help prepare the analysis pipelines with their own VWM tasks.

## EXPECTED RESULTS

Throughout this paper, we mention how procedures should be adjusted for particular protocols and participant cohorts. Therefore, it is important to understand how learning should occur when examining these groups. The below examples are from published data and cover the previously outlined protocols, as well as examples of data from healthy adults, older adults, and patient groups. We hope that these can provide a frame of reference for new researchers, who may be unsure if their data represent data typically recorded from certain cohorts of human participants.

### Protocols

**Figure 3:**
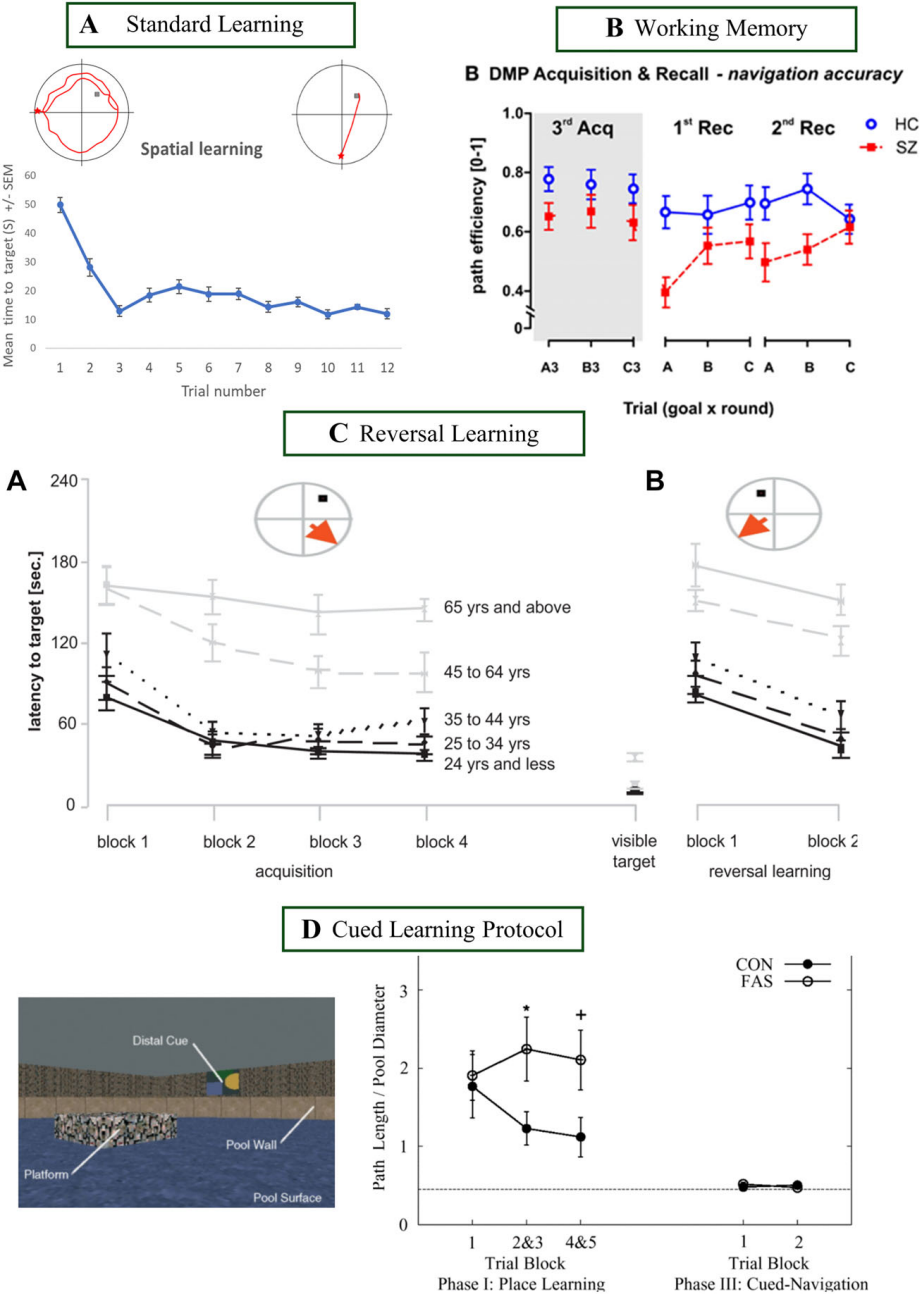
Examples of learning curves from differing protocols in healthy adults, including spatial learning **(A)** (Commins et al., 2020), working memory in healthy controls (HC) and schizophrenic patients (SZ) **(B)** (Fajernová et al., 2014), reversal learning **(C)** (Schoenfeld et al., 2014), and a cued‐learning protocol in control patients (CON) and those with fetal alcohol syndrome (FAS) **(D)** (Hamilton et al., 2003).

Figure 3A shows a typical *spatial learning* curve in which the escape latency decreases rapidly across trials (Commins et al., 2020; Woolley et al., 2010). The typical paths (see inset) go from random and/or thigmotaxis‐like behavior (hugging the side) through a direct path to the hidden target (small grey square in the NE quadrant). Figure 3B shows typical results from a *working memory* experiment with schizophrenic patients (SZ) and healthy controls (HC) (Fajnerová et al., 2014). In this example, there are 3 learning blocks of 3 trials (3^rd^ Acq), each block with a goal in different positions (A, B, and C). Participants are then asked to navigate to the goal locations in their original sequence (1^st^ Rec) and then complete a classic probe trial (2^nd^ Rec). Working memory in SZ is poorer compared to that of HC. In a *reversal learning* experiment (Figure 3C), initially, participants (especially younger adults, indicated by black lines) show good learning with a decrease in time to reach the target (left panel); the target is located in the NE quadrant. Then, following the change in platform location (platform now in the NW quadrant; see inset), the time to reach the new target increases for the first block before reducing again for the second block (right panel). Again, this is especially observed for younger adults. Finally, in a *cued‐learning* experiment, participants with fetal alcohol syndrome (FAS) were examined in a VWM by Hamilton et al. (2003). Deficits demonstrated in learning by the FAS participants compared to controls during place navigation (non‐cued; Phase I) were extinguished during cued trials (Phase III). This is a typical example of visible platform cued learning, which is generally efficient regardless of the population and makes for a useful control condition.

### Populations

**Figure 4:**
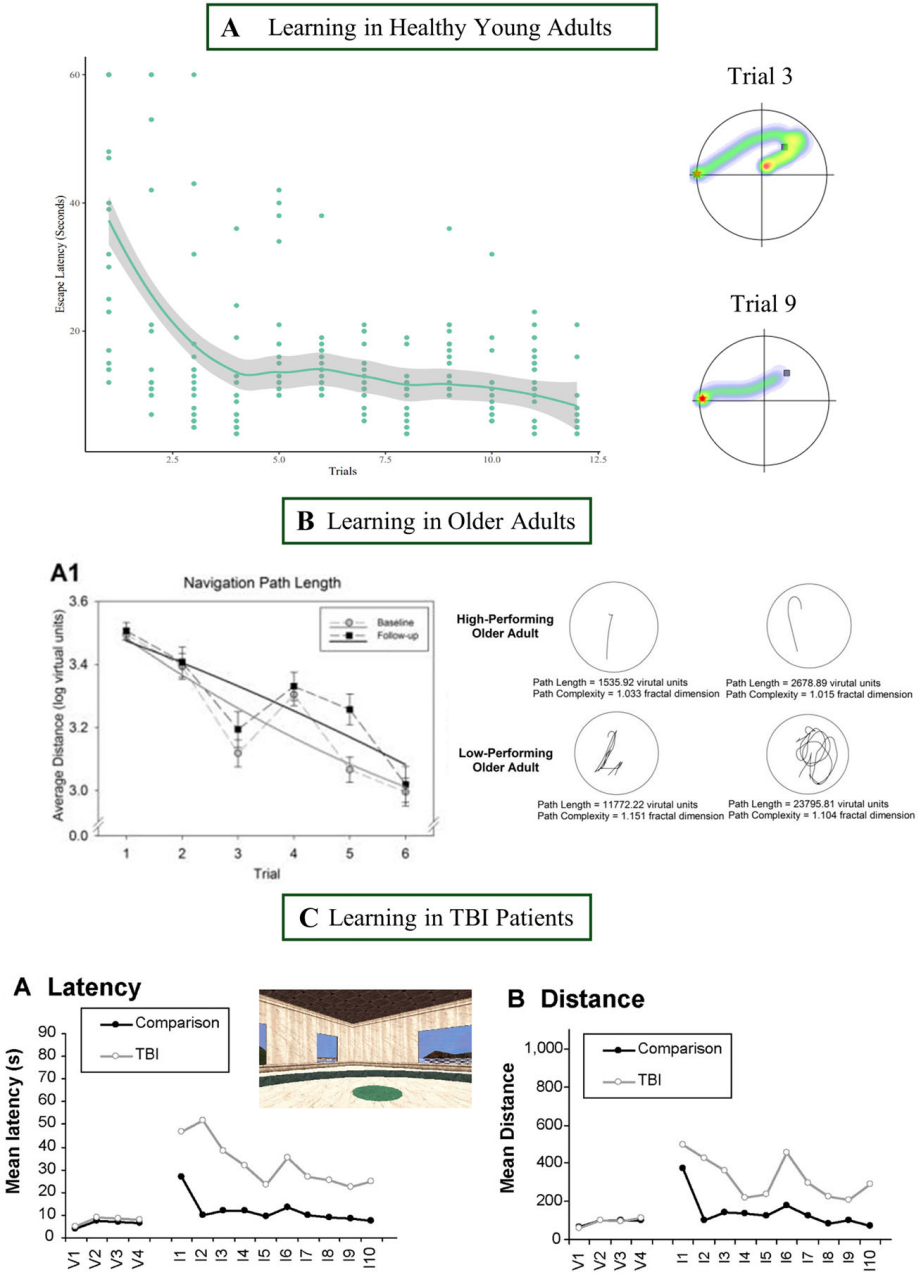
Examples of learning curves from different populations: younger adults **(A)** (sample data), older adults **(B)** (Daugherty & Raz, 2017), and patient population [traumatic brain injury (TBI)] **(C)** (Livingstone & Skelton, 2007).

**Figure 5:**
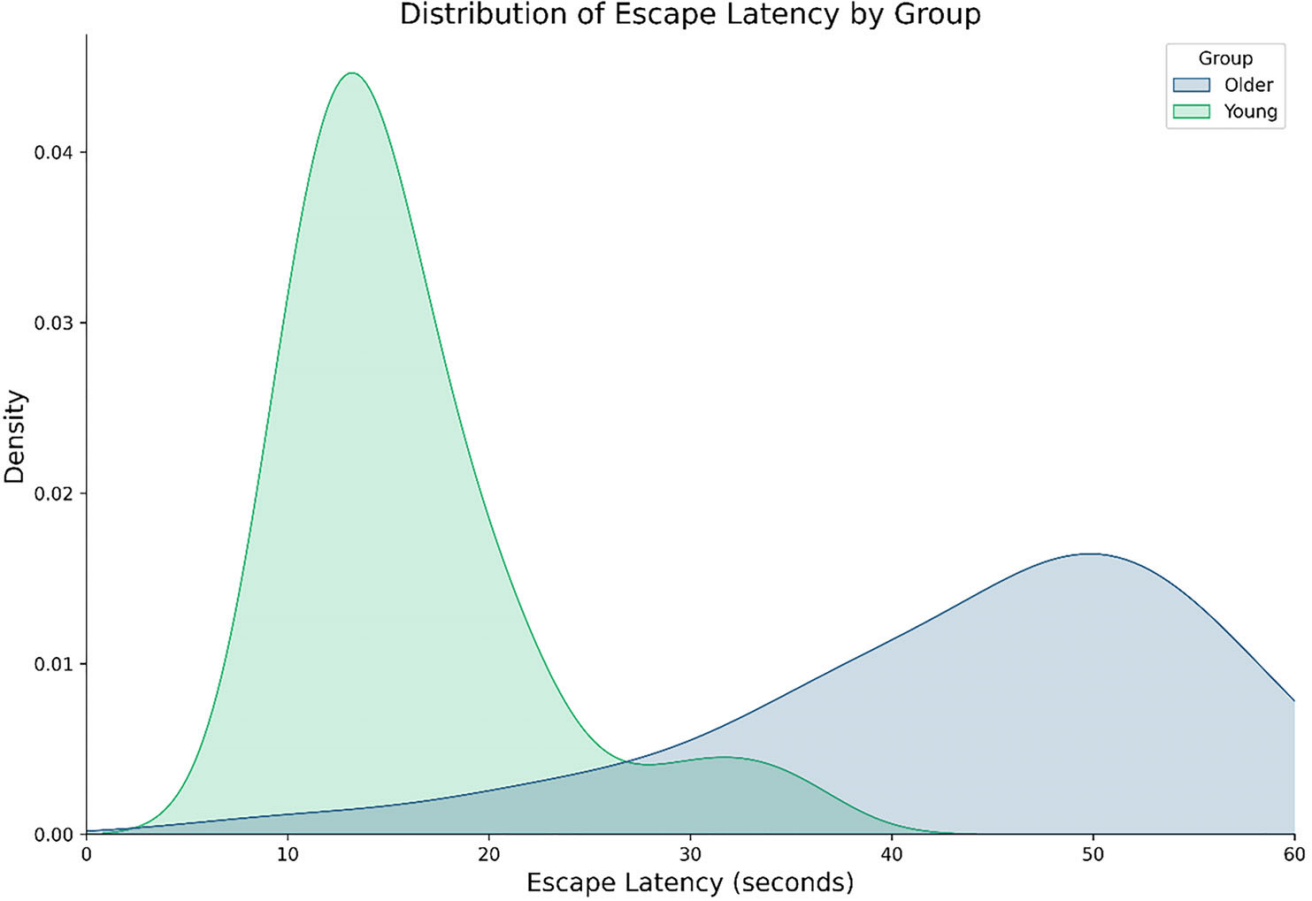
Probability density plot using kernel density estimation on sample datasets of older (n = 37) and younger (n = 44) adults who completed NavWell. The plot demonstrates the typical distribution (based on CI and SEM) in which our escape latency scores tend to fall in the majority of circumstances. This plot may be useful for comparison of escape latency scores across other software. Scores are cut‐off at 60 s maximum. The plot was generated using a short Python script involving the Matplotlib, Pandas, and Seaborn packages (Hunter, 2007; McKinney, 2010; Waskom, 2021). The script to reproduce the data (participant SEM and CI) and the plot are available at osf.io/ch5rn.

Figure 4A demonstrates a typical learning curve and path heatmaps (see inset) for healthy young adults, with escape latency decreasing rapidly across trials. Figure 4B demonstrates typical learning for older adults (Daugherty & Raz, 2017), with larger distances traveled, alongside fluctuations in performance across trials, with no asymptotic learning behavior. However, some older adults perform much better than others (see inset of path qualities), and it may be worth considering looking at *individual* learning ability when examining age (Commins et al., 2023). Therefore, we have provided a kernel probability density function of escape latency for older and younger adults (Figure 5). This provides a smoothed distribution of escape latency for age groups based on data from our own VWM, and we hope it will be useful for researchers to compare with their own datasets. Figure 4C displays comparative learning curves for a patient population (traumatic brain injury, TBI) and healthy age‐matched controls (Livingstone & Skelton, 2007). TBI patients perform worse than controls, taking longer to reduce escape latency and distance traveled during place‐learning trials (I1 to I10). However, it should be noted that deficits are not demonstrated in visible platform trials (V1 to V4).

## Author Contributions

**Conor Thornberry**: Conceptualization; investigation; writing—original draft; methodology; validation; visualization; writing—review and editing; software; formal analysis; project administration. **Jose M. Cimadevilla**: Writing—review and editing; conceptualization; validation. **Sean Commins**: Writing—review and editing; software; project administration; conceptualization; supervision.

## Conflict of Interest

The authors have no conflict of interest to disclose.

## Data Availability

The data that support the findings of this study are openly available in OSF at https://www.osf.io/ch5rn.
